# Important oncogenic and immunogenic roles of SPP1 and CSF1 in hepatocellular carcinoma

**DOI:** 10.1007/s12032-023-02024-7

**Published:** 2023-04-25

**Authors:** Tianxin Xiang, Na Cheng, Bo Huang, Xujun Zhang, Ping Zeng

**Affiliations:** 1 Department of Hospital Infection Control, The First Affiliated Hospital of Nanchang University, 17 Yongwai Road, Donghu District, Nanchang, China; 2 Department of Gynecology and Obstetrics, The First Affiliated Hospital, Zhejiang University School of Medicine, Hangzhou, China; 3 Hangzhou Normal University School of Basic Medical Sciences, Hangzhou, China; 4 Department of Radiology, Sir Run Run Shaw Hospital, Zhejiang University School of Medicine, No. 3, Qingchun East Road, Hangzhou, Zhejiang China; 5 Department of Hospital Infection Control, The First Affiliated Hospital of Nanchang University, Nanchang, China

**Keywords:** SPP1, CSF1, Hepatocellular Carcinoma, Oncogene, Interaction

## Abstract

The treatment and prognosis of liver cancer remain the focus of medical research. Studies have shown that SPP1 and CSF1 play important roles in cell proliferation, invasion, and metastasis. Therefore, this study analyzed the oncogenic and immunologic roles of SPP1 and CSF1 in hepatocellular carcinoma (HCC). We found that the expression levels of SPP1 and CSF1 in HCC were markedly increased and positively correlated. High SPP1 expression was significantly associated with poor OS, DSS, PFS, and RFS. It was not affected by gender, alcohol use, HBV, or race, whereas CSF1 was affected by these factors. Higher expression levels of SPP1 and CSF1 indicated higher levels of immune cell infiltration and a higher immune score with the R software package ESTIMATE. Further analysis revealed that many genes work co-expressed between SPP1 and CSF1 with the LinkedOmics database, which were mainly involved in signal transduction, the integral components of the membrane, protein binding, and osteoclast differentiation. In addition, we screened ten hub genes using cytoHubba, among which the expression of four genes was significantly associated with the prognosis of HCC patients. Finally, we demonstrated the oncogenic and immunologic roles of SPP1 and CSF1 using the vitro experiments. Reducing the expression of either SPP1 or CSF1 could significantly reduce the proliferation of HCC cells and the expression of CSF1, SPP1, and the other four hub genes. This study suggested that SPP1 and CSF1 interact with each other and have the potential to be therapeutic and prognostic targets for HCC.

**Supplementary Information:**

The online version contains supplementary material available at 10.1007/s12032-023-02024-7.

## Introduction

According to the latest global cancer statistics published by the International Agency for Research on Cancer in 2020, more than 900,000 new cases were diagnosed, and more than 830,000 patients died from liver cancer. The incidence rate ranks sixth, and the death rate ranks third in the worldwide [1]. HCC is the most common liver cancer, accounting for 90% of primary liver cancers, and HCC is one of the most serious threats to human health. Early diagnosis and treatment are crucial to prevent progression and reduce recurrence. However, due to the absence of early-stage symptoms of HCC, most patients are already in the advanced stage and past the optimum time for surgical treatment when diagnosed [2, 3]. Thus, further exploring the mechanism of HCC and finding key biomarkers for early diagnosis and new therapeutic strategies is important.

Secreted phosphoprotein 1 (SPP1, also named osteopontin, OPN), is an extracellular-matrix protein that is differentially expressed in a variety of cell types including osteoclasts, fibroblasts, epithelial cells, immune cells, lymphocytes, and cancer cells [4]. SPP1 is involved in the regulation of many tumor-associated biological processes, including cell proliferation and migration, tumorigenesis and progression. High SPP1 expression in tumor tissue was thought to be associated with poor prognosis in many types of cancers [5, 6]. This result is consistent with our previous study. Specifically, we revealed that SPP1 was a potential prognostic and immunotherapy biomarker in multiple human cancers using pan-cancer analysis [7]. However, the role of SPP1 in liver cancer warrants further investigation.

Colony-stimulating factor-1 (CSF1), also known as macrophage colony-stimulating factor, acts as a key cytokine in the production, recruitment, and activation of tumor-associated macrophages (TAMs) by binding with CSF1 receptor tyrosine kinase (CSF1R) [8]. CSF1 also plays an important role in many cancer types [9]. In primary colorectal cancer, the expression levels of CSF1 and CSF1R were associated with poor patient survival. CSF1R activation induced the epithelial-mesenchymal transition, invasion, migration, and metastasis of colorectal cancer cells via the STAT3-mediated downregulation of miR-34a [10]. In liver cancer, HCC-derived CSF1 transforms macrophages to the M2 phenotype to drive immune escape and anti-PD1 tolerance [11].

An increasing number of research articles have shown that SPP1 and CSF1 exhibit the same expression trend in multiple cancer types, including liver cancer, breast cancer, thyroid cancer, and lung cancer [12–15]. In our previous study, SPP1 was identified as a potential marker for prognostic assessment in multiple cancers. OPN was recently reported to be able to block the Th1-mediated immune response pathway by activating the colony-stimulating factor-1 (CSF1)/CSF1 receptor (CSF1R) pathway, leading to an increase in immunosuppressive cytokines in liver cancer [12]. Therefore, the roles of SPP1 and CSF1 in HCC and their relationship warrant investigation.

## Materials and methods

### Expression and prognostic value analysis of SPP1 and CSF1 in liver hepatocellular carcinoma

We downloaded the TCGA expression profile and corresponding visualization of SPP1 and CSF1 containing multiple human normal and tumor tissues from the Sangerbox database (http://www.sangerbox.com/Tool). Then, the UALCAN database (http://ualcan.path.uab.edu) [16] was used for subgroup analysis of SPP1 and CSF1 expression, including sample types, individual cancer stages, tumor grade, nodal metastasis status, TP53 mutation status, tumor histology, patient’s race, gender, age, and weight. Then, the survival value of SPP1 and CSF1 in liver cancer was performed by the Kaplan–Meier Plotter database (http://kmplot.com) including overall survival (OS), disease-specific survival (DFS), relapse-free survival (RFS), and progression-free survival (PFS). The gene expression correlation analysis of SPP1 and CSF1 was conducted using the Gene Expression Profiling Interactive Analysis (GEPIA) database (http://gepia2.cancer-pku.cn/).

### Correlation analysis of SPP1 expression and Immune cell infiltration

We performed the correlation analysis of SPP1 and CSF1 expression and immune cells using the Tumor Immune Estimate Resource (TIMER) database (http://cistrome.shinyapps.io/timer/) [17]. The transcriptome RNA-seq data of liver cancer was downloaded from the University of California Santa Cruz (UCSC) Xena website (http://xena.ucsc.edu/) [18]. The ESTIMATE immune score was used to estimate the infiltration levels of immune cells by R software package (R packages, version 3.6.3) ESTIMATE (https://R-Forge.R-project.org/projects/estimate/).

### Differential and co-expressed gene analysis in HCC

We analyzed differential expressed genes correlated with SPP1 and CSF1 using the LinkedOmics database (http://www.linkedomics.org/login.php) [19]. The correlation analysis used Spearman tests. The volcano and heatmap were used to display the analysis results. The co-expression gene was screened by setting a correlation coefficient > 0.4 and the corresponding visualization was performed using the Venn graph.

### Protein–protein interaction (PPI) network, Hub gene analysis and function analysis

The PPI network was constructed using the STRING database (http://string-db.org) [20]. The hub gene analysis was conducted by the cytoHubba app in Cytoscape software (version 3.8.2) [21]. Then, functional analysis of gene was performed with the Database for Annotation, Visualization, and Integrated Discovery (DAVID) Bioinformatics Resources 6.8 (https://david.ncifcrf.gov/home.jsp) [22] including Gene Ontology (GO) analysis and Kyoto Encyclopedia of Genes and Genomes (KEGG) pathway enrichment analysis. The hub gene expression and correlation analysis with SPP1 were conducted using the Gene Expression Profiling Interactive Analysis (GEPIA) database (http://gepia2.cancer-pku.cn/) [23]. In addition, we compared gene expression between TCGA tumor samples and normal samples in TCGA liver cancer (496 samples) from the UCSC Xena website.

### Cell proliferation assays

The human cell lines SNU499 and Hep3B (hepatoma carcinoma) were purchased from the Cell Bank of the Chinese Academy of Sciences. EdU staining was used for the detection of cell proliferation. Briefly, cells were seeded in 24-well plates at a density of 2 × 10^3^ cells/well. EdU kit (Beyotime, Shanghai, China) was used for detecting cell proliferation according to the manufacturer’s instruction.

### Quantitative real-time PCR (qRT-PCR)

Total RNA was isolated using a Trizol reagent (Takara). cDNA was synthesized using a PrimeScriptTM RT Msater Mis (Takara). qRT-PCR analyses were conducted with SYBR® Premix Ex TaqTM II (Takara) with specific primers as follows:

**Table:** 

SPP1:	5′-CTCCATTGACTCGAACGACTC-3′ (Forward),
	5′-CAGGTCTGCGAAACTTCTTAGAT-3′ (Reverse);
CSF1:	5′-TGGCGAGCAGGAGTATCAC-3′ (Forward),
	5′-AGGTCTCCATCTGACTGTCAAT-3′ (Reverse);
CLEC5A:	5′-AGGTGGCGTTGGATCAACAA-3′ (Forward),
	5′-TTAGGCCAATGGTCGCACAG-3′ (Reverse);
GPR84:	5′-GTGCTGGGCTATCGTTATGTT-3′ (Forward),
	5′-GAATCGGGTACGGAGCTTGG-3′ (Reverse);
ITGAV:	5′-ATCTGTGAGGTCGAAACAGGA-3′ (Forward),
	5′-TGGAGCATACTCAACAGTCTTTG-3′ (Reverse);
PLAUR:	5′-TGTAAGACCAACGGGGATTGC-3′ (Forward),
	5′-AGCCAGTCCGATAGCTCAGG-3′ (Reverse).

### Statistical analysis

R software (version 3.6.3) or Perl software (Strawberry Perl 5.30.0.1 64-bit) was performed analyses and chart visualize in this study. Statistical analysis of gene different expression was performed by using GraphPad Prism version 8.0. P-value < 0.05 were considered statistically significant.

## Results

### Overexpression of SPP1 and CSF1 in HCC

**Fig. 1:**
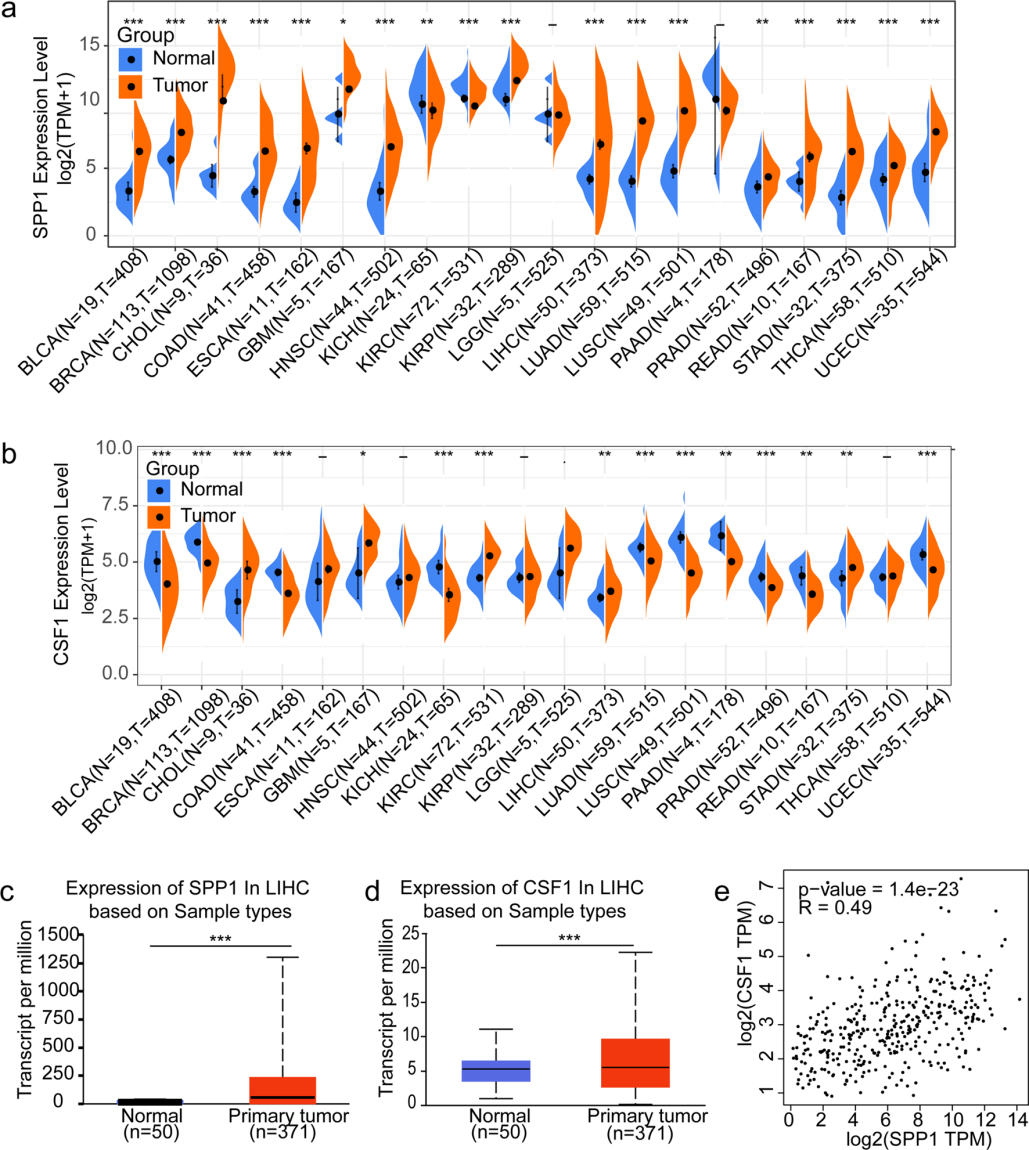
SPP1 and CSF1 expression levels in different cancer types. **a** SPP1 expression in different tumor tissues compared with that in normal tissues. **b** CSF1 expression in different tumor tissues compared with that in normal tissues. **c** SPP1 expression in HCC. **d** CSF1 expression in HCC. **e** Correlation between SPP1 and CSF1 in HCC. (*P < 0.05, **P < 0.01, ***P < 0.001)

The graphical abstract shows the overall design of the study. To investigate the expression levels of SPP1 and CSF1 in tumor and normal tissues from cancer cases, we used the Sanger database and found that the mRNA expression levels of SPP1 and CSF1 were significantly increased in multiple cancer types (cancer vs. normal), such as CHOL (cholangiocarcinoma), ESCA (esophageal carcinoma), GBM (glioblastoma multiforme), LIHC (liver hepatocellular carcinoma), and STAD (stomach adenocarcinoma) **(**Fig. 1a, b**)**. To increase the reliability of the results, an analysis of the UALCAN database again validated that SPP1 and CSF1 were highly expressed in liver cancer **(**Fig. 1c, d**)**. In addition, the expression levels of SPP1 and CSF1 were positively correlated **(**Fig. 1e**)**. To further investigate the mRNA expression levels of SPP1 and CSF1 in different liver cancer samples from the TCGA database, we performed a subgroup analysis using the UALCAN database. Compared with normal samples, the expression levels of SPP1 and CSF1 were higher in subgroup cancer samples, including subgrouping by gender, age, weight, race, stage, tumor grade, nodal metastasis status, and TP53 mutant status (Fig. S1). In general, these results indicated that SPP1 and CSF1 were highly expressed in HCC.

### The prognostic value of SPP1 and CSF1 in HCC

We hypothesized that the expression levels of SPP1 and CSF1 were related to the prognosis of liver cancer patients. Therefore, we performed a meta-analysis to validate the survival biomarkers using the Kaplan‒Meier Plotter database. In HCC patients (n = 364), higher gene expression was associated with a lower survival probability (OS: HR = 2.27, log-rank P = 3.5e-06; DSS: HR = 2.03, log-rank p = 0.0016; PFS: HR = 1.59, log-rank p = 0.0017; RFS: HR = 1.76, log-rank P = 0.00069) (Fig. S2a–d). To further verify the survival significance of SPP1 in different liver cancer patients, a subgroup analysis was also conducted. The results showed that race, gender, hepatitis virus, and alcohol consumption did not affect the correlation between SPP1 and survival (Fig. S2e-l). We also analyzed the prognostic value of CSF1. The results showed that high CSF1 expression was associated with poor OS (HR = 1.69, log-rank P = 0.0043) and was not associated with PFS, RFS, or DSS (Fig. S3a–d). In addition, high CSF1 expression was also associated with poor OS in liver cancer patients who were male, did not consume alcohol, had HBV, and were Asian (Fig. S3e-l). Taken together, SPP1 and CSF1 could be potential prognostic markers for HCC patients.

### Correlation of SPP1 and CSF1 expression with the infiltration of immune cells in HCC

**Fig. 2:**
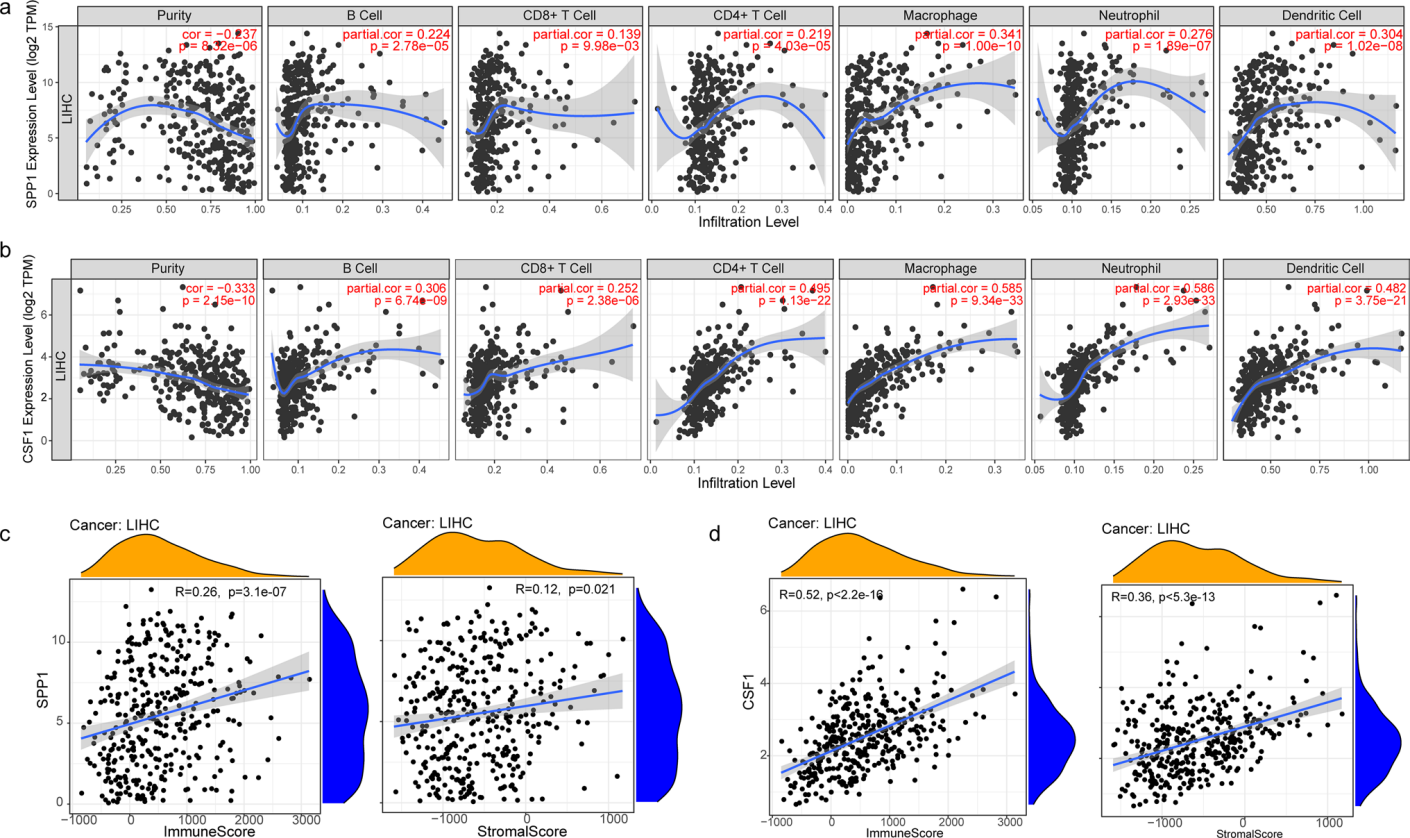
Correlation of SPP1 and CSF1 with immune infiltration in HCC. **a** Correlation between SPP1 expression and immune infiltration cells. **b** Correlation between CSF1 expression and immune infiltration cells. **c** Correlation between SPP1 expression and immune/stromal scores. **d** Correlation between CSF1 expression and immune/stromal scores

Immune cell infiltration is correlated with tumor progression and prognosis. Hence, the correlation of SPP1 and CSF1 expression with immune cell infiltration was explored using the TIMER database. The expression of SPP1 was negatively associated with tumor purity (cor = -0.237, p = 8.32e-06) and positively associated with immune cells, including B cells (cor = 0.224, p = 2.78e-06), CD8 + T cells (cor = 0.139, p = 9.98e-03), CD4 + T cells (cor = 0.219, p = 4.03e-05), macrophages (cor = 0.341, p = 1.00e-10), neutrophils (cor = 0.276, p = 1.89e-07) and dendritic cells (cor = 0.304, p = 1.28e-08)** (**Fig. 2a**)**. The correlation between CSF1 expression and immune cell infiltration also showed the same trend** (**Fig. 2b**)**. Immune and stromal scores could contribute to determining tumor purity and immune cell infiltration in the tumor microenvironment. A higher score indicates more immune cells and stromal cells, which consequently indicates a lower the tumor purity. The expression of SPP1 in liver cancer patients was positively correlated with the immune score (R = 0.26, p = 3.1e-07) and stromal score (R = 0.12, p = 0.021) (Fig. 2c). The expression of CSF1 was also positively correlated with immune score (R = 0.52, p < 2.2e-16) and stromal score (R = 0.36, p < 5.3e-13) (Fig. 2d). The above results indicate that the expression levels of SPP1 and CSF1 positively correlated with the malignancy of tumors.

### Co-expressed genes and functional analysis of SPP1 and CSF1 in HCC

**Fig. 3:**
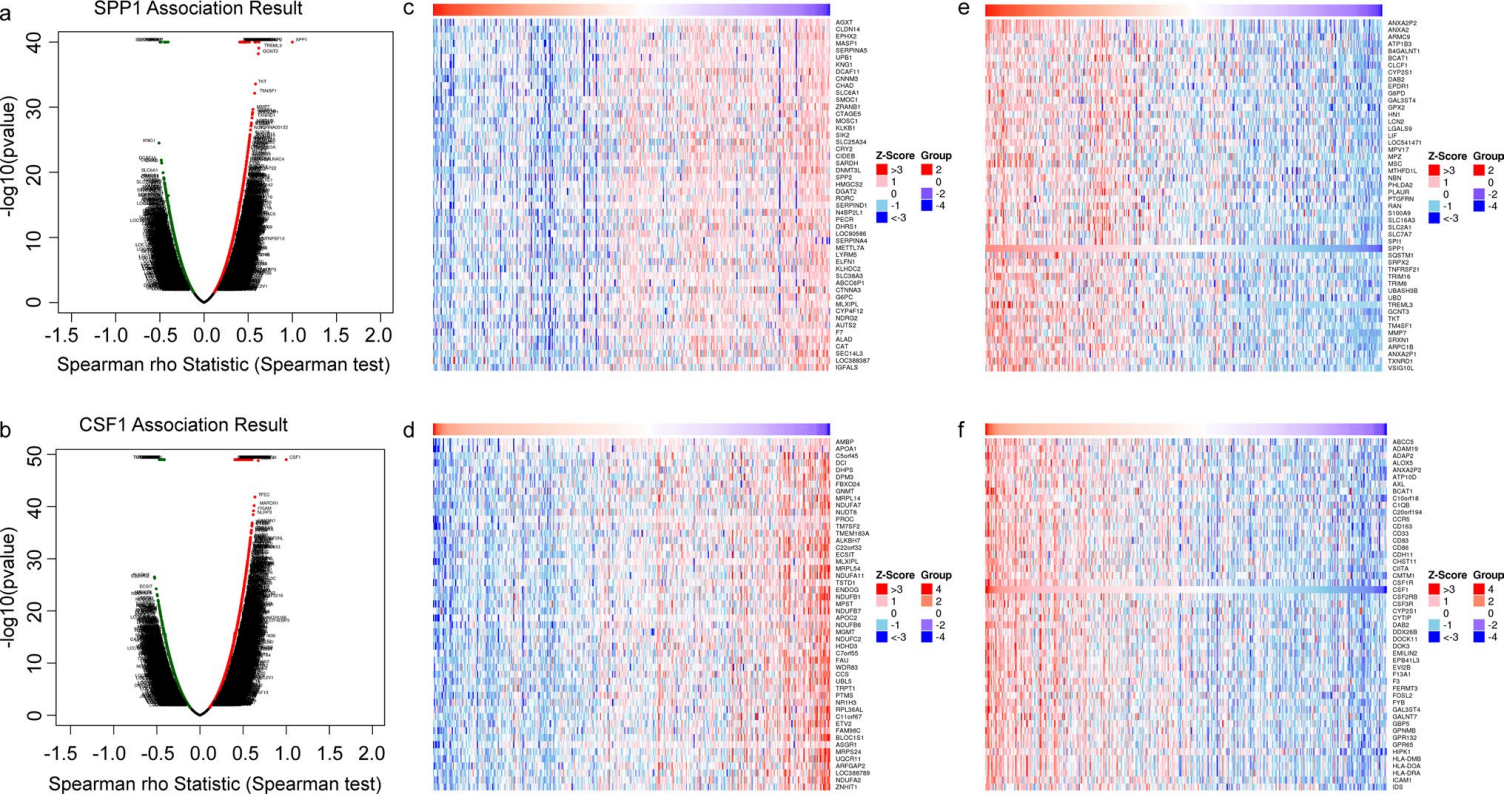
Differential genes of SPP1 and CSF1 association in HCC. **a** Volcano plot showing the differentially expressed genes of SPP1. (b) Volcano plot showing the differentially expressed genes of CSF1. **c, e** Heatmap showing that the genes are positively and negatively correlated with SPP1 (only the top 50 genes are shown). **d, f** Heatmap showing that the genes are positively and negatively correlated with CSF1 (only top 50 genes are shown)

**Fig. 4:**
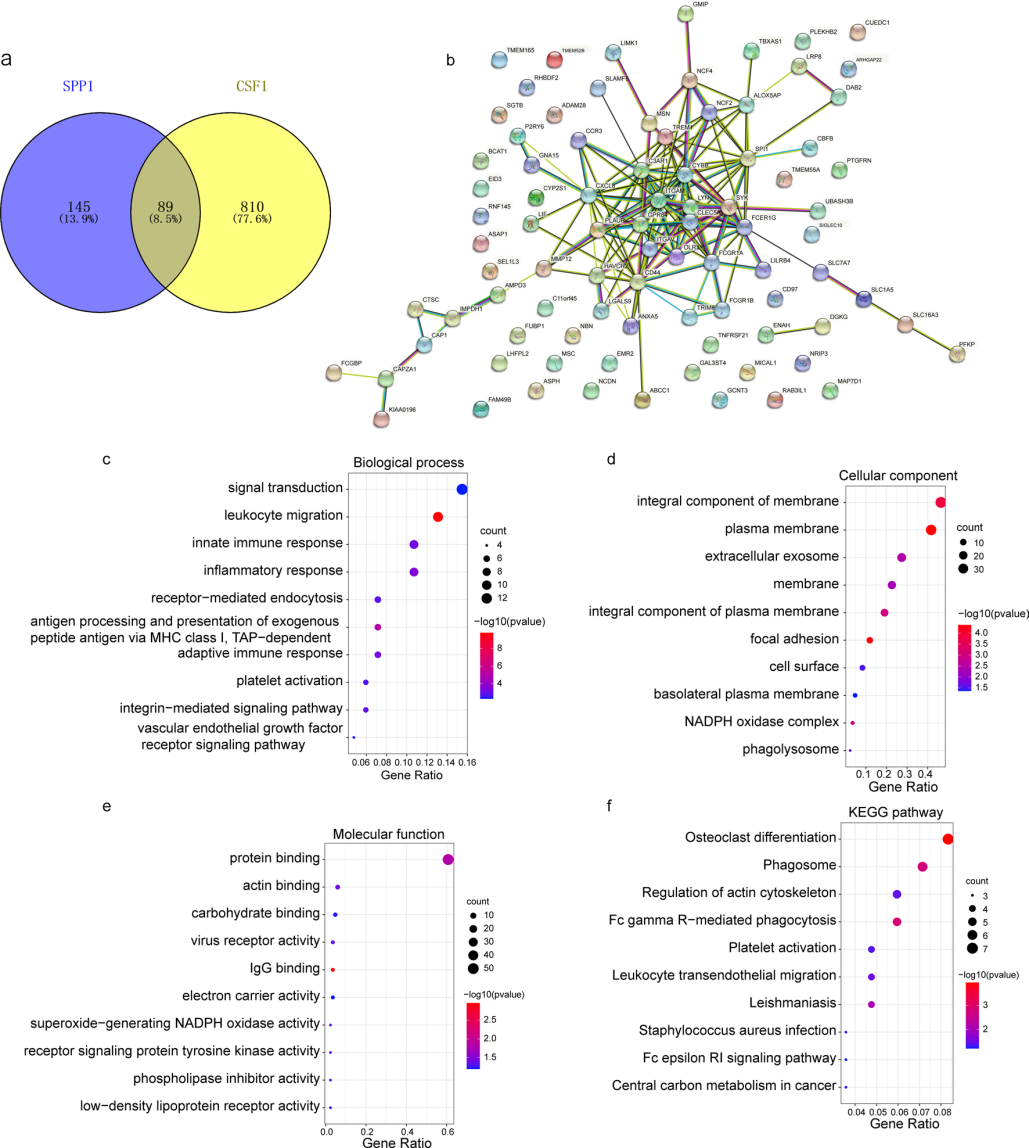
Analysis of co-expressed genes of SPP1 and CSF1 and their functional analysis. **a** Venn diagram showing the co-expression of SPP1 and CSF1. **b** The interaction network of the 89 co-expressed genes. **c–f** GO and KEGG enrichment analyses of the 89 co-expressed genes

To identify differentially expressed genes associated with SPP1 and CSF1 in HCC, we performed a Spearman test using the LinkedOmics database. The volcano map showed differentially expressed genes **(**Fig. 3a, b**)**. The top 50 negatively and top 50 positively correlated genes are displayed in a heatmap **(**Fig. 3c–f**)**. We selected the positively correlated genes with coefficients > 0.4 based on the Spearman test. In total, 234 genes positively correlated with SPP1 and 899 genes positively correlated with CSF1. Among these genes, we found that 89 genes showed positive correlations with both SPP1 and CSF1 **(**Fig. 4a**)**. We constructed a PPI network based on these genes using STRING and Cytoscape and found that most genes interacted with each other **(**Fig. 4b**)**. To further understand the function of these genes, GO and KEGG enrichment analyses were performed using the DAVID database. The biological process terms were significantly affected in signal transduction, leukocyte migration, innate immune response, and inflammatory response **(**Fig. 4c**)**. The cellular component terms were mainly enriched in the integral component of the membrane, plasma membrane, and extracellular exosome **(**Fig. 4d**)**. The molecular function terms were mainly enriched in protein binding, actin binding, and carbohydrate-binding **(**Fig. 4e**)**. The KEGG results showed that the differentially expressed genes were mainly involved in osteoclast differentiation, the phagosome, and the regulation of actin cytoskeleton **(**Fig. 4f**)**. We also specifically analyzed the role of the SPP1 and CSF1 signaling pathways in HCC using the LinkOmics database. The results showed that SPP1 and CSF1 participated in many of the same signaling pathways, including the NF-kappa B signaling pathway, TNF signaling pathway, and PPAR signaling pathway (Fig. S4). Based on the above results, we believe that SPP1 and CSF1 interact with each other and play important roles in the same signaling pathway.

### Screening of hub genes and their prognostic value in HCC

**Fig. 5:**
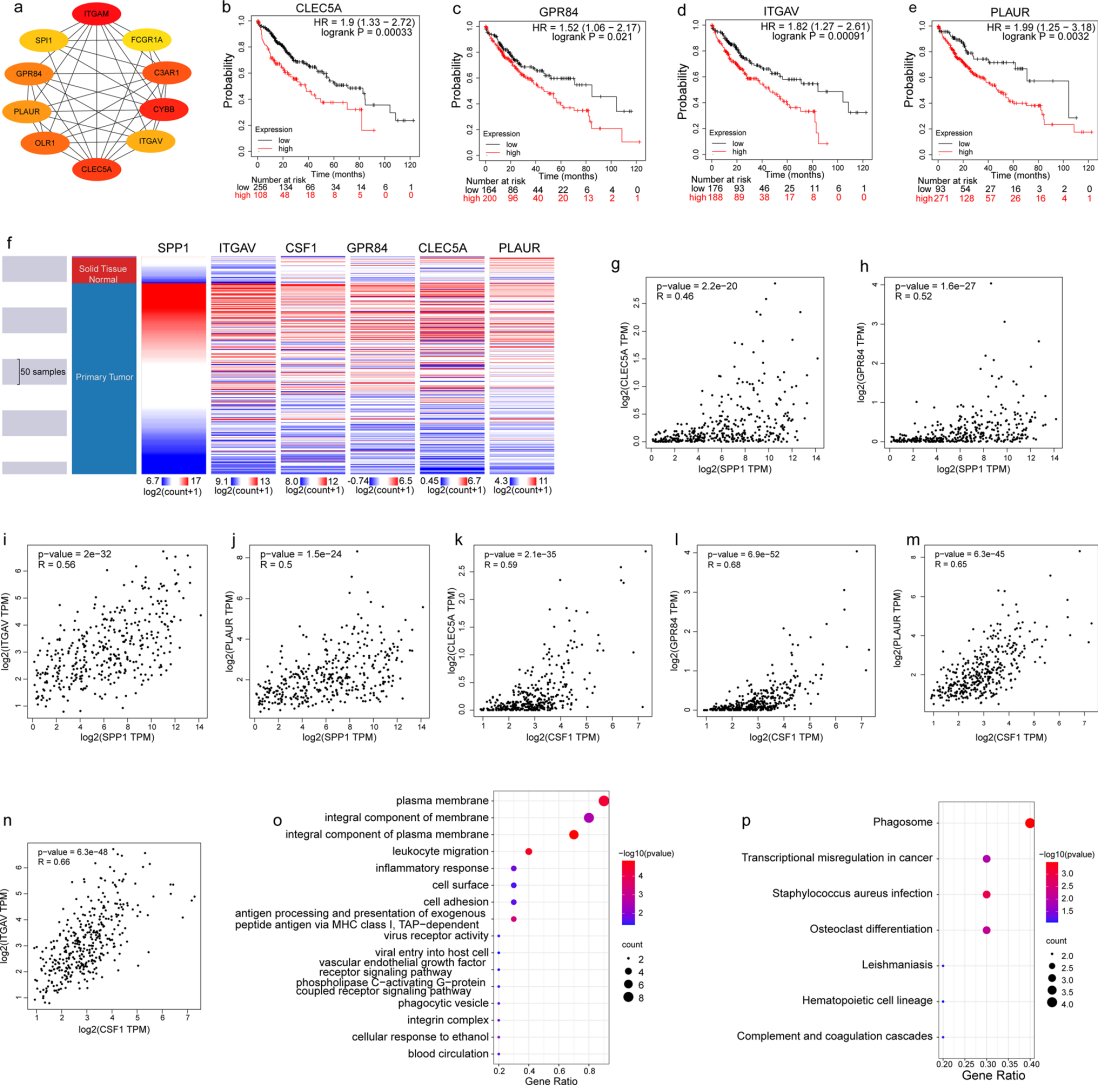
Hub gene analysis. **a** The interaction network of the top 10 hub genes. **b–e** The prognostic value of four hub genes with significant differences (CLEC5A, GPR84, ITGAV, PLAUR) (P < 0.05). **f** The expression pattern of SPP1, CSF1 and four hub genes. **g–j** Correlation of SPP1 with four hub genes. **k–n** Correlation of CSF1 with four hub genes. **o, p** Functional analysis of the top 10 hub genes

We used cytoHubba (ranked by degree) to screen the top ten hub genes of the network, including CLEC5A, GPR84, ITGAV, PLAUR, SPI1, FCGR1A, C3AR1, CYBB, OLR1, and ITGAM **(**Fig. 5a**)**. The prognostic value of the hub genes was evaluated using Kaplan‒Meier Plotter. We found that high expression levels of four genes were significantly related to poor OS (CLEC5A, GPR84, ITGAV, and PLAUR)** (**Fig. 5b–e**)**, while the other six genes were not significantly related to survival (SPI1, FCGR1A, C3AR1, CYBB, OLR1, and ITGAM)** (Fig. S5)**. We also compared TCGA tumor samples to GTEx normal samples to determine whether SPP1 and CSF1 were up- or downregulated in HCC. The results showed that the expression patterns of SPP1 and CSF1 in normal and tumor tissues were most similar to those of ITGAV and PLAUR, respectively **(**Fig. 5f**)**. In addition, we further analyzed the correlation between the expression levels of SPP1 and CSF1 and the hub genes. The results showed that SPP1 and CSF1 significantly correlated with the four hub genes** (**Fig. 5g–n**)**. GO analysis results were significantly affected and enriched in the plasma membrane, integral component of membrane, integral component of plasma membrane, and leukocyte migration domains **(**Fig. 5o**)**. KEGG analysis results were significantly affected and enriched in the phagosome, transcriptional misregulation in cancer, Staphylococcus aureus infection, and osteoclast differentiation domains **(**Fig. 5p**)**.

### Validation of the interaction between SPP1 and CSF1 in HCC cell lines

**Fig. 6:**
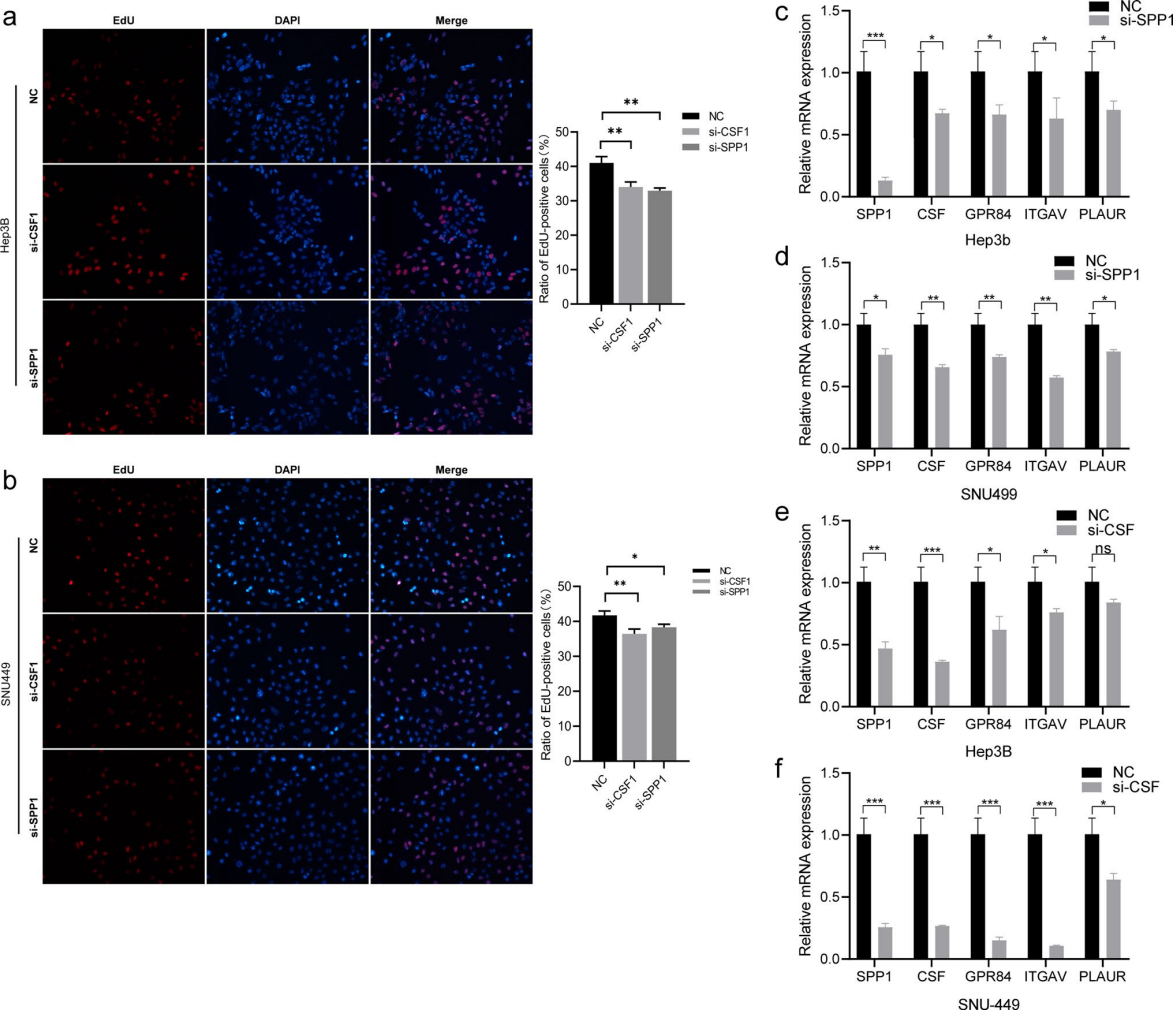
Function and interaction of SPP1 and CSF1. **a** Cell proliferation tested by EdU assay after siRNA-SPP1 or siRNA-CSF1 in Hep3B cells. **b** Cell proliferation tested by EdU assay after siRNA-SPP1 or siRNA-CSF1 in SNU449 cells. **c, d** The mRNA expression of SPP1, CSF1 and four hub genes after siRNA-SPP1 in Hep3B and SNU449 cells. (e, f) The mRNA expression of SPP1, CSF1 and four hub genes after siRNA-CSF1 in Hep3B and SNU449 cells. (*P < 0.05, **P < 0.01, ***P < 0.001)

To further verify the interaction between SPP1 and CSF1, we analyzed the interaction between SPP1 and CSF1 in hepatoma cell lines. We used siRNA transfection to knockdown SPP1 or CSF1 expression in Hep3B and SNU499 cell lines. The results of EdU staining assays showed that si-SPP1 and si-CSF1 decreased tumor cell proliferation **(**Fig. 6a, b**)**. We also measured hub gene expression. The results showed that si-SPP1 and si-CSF1 could downregulate the expression of these genes in Hep3B and SNU499 cell lines, but to different degrees **(**Fig. 6c–f**)**.

### Discussion

Notably, liver cancer continues to be a major challenge in public health worldwide, with high mortality and poor prognosis. Although multiple therapeutic methods, such as surgery, radiotherapy, chemotherapy, and liver transplantation, are available, the efficacy of treatments remains limited. Due to a lack of precise diagnostic measures and the hallmarks of HCC, patients are often diagnosed at an advanced stage and lose the best treatment chance. Therefore, exploring the molecular biological regulatory network and screening prognostic biomarkers is of great significance for the individualized treatment of liver cancer patients.

The expression of SPP1 is significantly increased and related to poor prognosis in multiple cancer types, while decreasing SPP1 expression inhibits the proliferation, migration and invasion of cancers [7, 24]. In melanoma patients, SPP1 expression was positively correlated with melanoma progression, and higher SPP1 expression indicated a worse prognosis, whereas silencing SPP1 suppressed melanoma cell proliferation, and migration [25]. In breast cancer, the level of SPP1 was significantly higher in tumors than in adjacent tissues and was positively correlated with tumor grade and subtype [26]. In gastric cancer cell lines, the elevated expression of SPP1 was a critical determinant of poor prognosis [27]. In HCC, the expression of SPP1 mRNA and OPN protein increased and correlated with poor outcomes in HCC patients [28]. In our study, the expression trend of CSF1 was not consistent across cancer types. CSF1 was highly expressed in bile duct, brain, kidney, liver, and stomach cancers, while it was expressed at low levels in bladder, breast, colon, lung, pancreatic, prostate, and uterine cancers. In human melanoma, the expression of CSF1 in blood was significantly higher in metastatic melanoma than in healthy subjects [29]. However, studies have shown that high CSF1 expression in tumors was also associated with poor prognosis [30]. High expression levels of CSF1 and CSF1R were related to breast cancer progression and poor prognosis [31]. Zhu et al. showed that CSF1 expression increases in invasive carcinoma compared to normal pancreatic tissue [32]. Wang et al. found that the overexpression of CSF1 in colon cancer cells was correlated with macrophage infiltration and positively associated with the survival of colon cancer patients [33]. Therefore, further studies are needed to determine the role of CSF1 in tumors.

OPN is a critical driver of immune escape in the HCC tumor microenvironment. As an immune-related gene, CSF1 is also involved in the tumor immune microenvironment and stimulates the growth and survival of myeloid-derived suppressor cells and other myeloid-lineage cells [34]. CSF1 plays an important role in the proliferation, differentiation, and survival of monocyte/macrophage-lineage cells by binding to CSF1R, which might promote their acquisition of an immunosuppressive M2-like phenotype [35]. CSF1 expression correlated with the abundance of CD8 T cells and CD163 tumor-associated macrophages (TAMs) in melanoma. Adaptive CSF1 secretion upon exposure to T-cell-derived cytokines may act detrimentally to recruit TAMs and consequently hamper antitumor immune responses [29]. The activation of the CSF1/CSF1R pathway could block the Th1-mediated immune response and other classical antitumor immune effector pathways, whereas blocking the CSF1/CSF1R pathway could prevent TAM trafficking and enhance the efficacy of immune checkpoint-based therapies in HCC. These processes were related to the expression level of OPN [12]. In addition, studies have shown that OPN secretion could mediate the immune complex activation of human monocytes to partially promote lung fibroblast migration, and CSF1 could amplify the effect of OPN [36]. In our study, the expression levels of SPP1 and CSF1 positively correlated with immune infiltrating cells. These studies indicated that SPP1 and CSF1 interact with each other during immune regulation.

In some tumor-related studies, SPP1 and CSF1 exhibit the same expression trend and function. In thyroid cancer, the expression levels of SPP1 and CSF1 are significantly up-regulated, while CSF1R inhibitor can reduce their expression, thereby inhibiting tumor cell proliferation [14]. In lung cancer, the expression levels of CSF1 and SPP1 are increased and associated with the carcinogenesis and prognosis of patients [15]. In breast cancer, the inhibition of SPP1 and CSF1 could prevent the assembly of an immunosuppressive tumor microenvironment and partially or completely sensitize otherwise refractory quasi-mesenchymal tumors to anti-CTL4 immune checkpoint blockade therapy [13]. In our study, the expression trend of SPP1 and CSF1 in HCC was consistent, which was verified using liver cancer cells in vitro.

## Conclusion

In summary, we found that SPP1 and CSF1 were highly expressed in HCC, and positively correlated with poor prognosis and immune cell infiltration, indicating that they have the potential to be therapeutic and prognostic targets for HCC. In addition, SPP1 and CSF1 have co-expressed genes and interact with each other. However, the specific mechanism needs to be further studied.

## Supplementary Information

Below is the link to the electronic supplementary material.

Supplementary file1 (DOCX 1043 KB)

## Data Availability

Publicly available datasets were analyzed in this article.

## References

[1] Siegel RL, Miller KD, Fuchs HE, Jemal A (2021). Cancer Statistics, 2021. CA Cancer J Clin.

[2] Mazzaferro V, Citterio D, Bhoori S, Bongini M, Miceli R, De Carlis L (2020). Liver transplantation in hepatocellular carcinoma after tumour downstaging (XXL): a randomised, controlled, phase 2b/3 trial. Lancet Oncol.

[3] Zhou T, Li S, Xiang D, Liu J, Sun W, Cui X (2020). m6A RNA methylation-mediated HNF3gamma reduction renders hepatocellular carcinoma dedifferentiation and sorafenib resistance. Signal Transduct Target Ther.

[4] Pang X, Gong K, Zhang X, Wu S, Cui Y, Qian BZ (2019). Osteopontin as a multifaceted driver of bone metastasis and drug resistance. Pharmacol Res.

[5] Tang H, Chen J, Han X, Feng Y, Wang F (2021). Upregulation of SPP1 Is a marker for poor lung cancer prognosis and contributes to cancer progression and cisplatin resistance. Front Cell Dev Biol.

[6] Wang Y, Zheng K, Chen X, Chen R, Zou Y (2021). Biosci Rep.

[7] Zeng P, Zhang X, Xiang T, Ling Z, Lin C, Diao H (2022). Secreted phosphoprotein 1 as a potential prognostic and immunotherapy biomarker in multiple human cancers. Bioengineered.

[8] Li X, Liu R, Su X, Pan Y, Han X, Shao C (2019). Harnessing tumor-associated macrophages as aids for cancer immunotherapy. Mol Cancer.

[9] Buechler MB, Fu W, Turley SJ (2021). Fibroblast-macrophage reciprocal interactions in health, fibrosis, and cancer. Immunity.

[10] Shi X, Kaller M, Rokavec M, Kirchner T, Horst D, Hermeking H (2020). Characterization of a p53/miR-34a/CSF1R/STAT3 Feedback Loop in Colorectal Cancer. Cell Mol Gastroenterol Hepatol.

[11] Wei CY, Zhu MX, Zhang PF, Huang XY, Wan JK, Yao XZ (2022). PKCalpha/ZFP64/CSF1 axis resets the tumor microenvironment and fuels anti-PD1 resistance in hepatocellular carcinoma. J Hepatol.

[12] Zhu Y, Yang J, Xu D, Gao XM, Zhang Z, Hsu JL (2019). Disruption of tumour-associated macrophage trafficking by the osteopontin-induced colony-stimulating factor-1 signalling sensitises hepatocellular carcinoma to anti-PD-L1 blockade. Gut.

[13] Dongre A, Rashidian M, Eaton EN, Reinhardt F, Thiru P, Zagorulya M (2021). Direct and indirect regulators of epithelial-mesenchymal transition-mediated immunosuppression in breast carcinomas. Cancer Discov.

[14] Park S, Kim M, Zhu J, Lee WK, Altan-Bonnet G, Meltzer P (2020). Inflammation suppression prevents tumor cell proliferation in a mouse model of thyroid cancer. Am J Cancer Res.

[15] Wang YD, Li Z, Li FS (2020). Differences in key genes in human alveolar macrophages between phenotypically normal smokers and nonsmokers: diagnostic and prognostic value in lung cancer. Int J Clin Exp Pathol.

[16] Chandrashekar DS, Bashel B, Balasubramanya SAH, Creighton CJ, Ponce-Rodriguez I, Chakravarthi B (2017). UALCAN: a portal for facilitating tumor subgroup gene expression and survival analyses. Neoplasia.

[17] Li T, Fan J, Wang B, Traugh N, Chen Q, Liu JS (2017). TIMER: a web server for comprehensive analysis of tumor-infiltrating immune cells. Cancer Res.

[18] Goldman M, Craft B, Hastie M, Repečka K, Kamath A, McDade F (2019). The UCSC Xena platform for public and private cancer genomics data visualization and interpretation. bioRxiv.

[19] Vasaikar SV, Straub P, Wang J, Zhang B (2018). LinkedOmics: analyzing multi-omics data within and across 32 cancer types. Nucleic Acids Res.

[20] Szklarczyk D, Gable AL, Lyon D, Junge A, Wyder S, Huerta-Cepas J (2019). STRING v11: protein-protein association networks with increased coverage, supporting functional discovery in genome-wide experimental datasets. Nucleic Acids Res.

[21] Chin CH, Chen SH, Wu HH, Ho CW, Ko MT, Lin CY (2014). cytoHubba: identifying hub objects and sub-networks from complex interactome. BMC Syst Biol.

[22] Dennis G, Sherman BT, Hosack DA, Yang J, Gao W, Lane HC (2003). DAVID: database for annotation, visualization, and integrated discovery. Genome Biol.

[23] Tang Z, Li C, Kang B, Gao G, Li C, Zhang Z (2017). GEPIA: a web server for cancer and normal gene expression profiling and interactive analyses. Nucleic Acids Res.

[24] Liu K, Hu H, Jiang H, Liu C, Zhang H, Gong S (2020). Upregulation of secreted phosphoprotein 1 affects malignant progression, prognosis, and resistance to cetuximab via the KRAS/MEK pathway in head and neck cancer. Mol Carcinog.

[25] Deng G, Zeng F, Su J, Zhao S, Hu R, Zhu W (2020). BET inhibitor suppresses melanoma progression via the noncanonical NF-kappaB/SPP1 pathway. Theranostics.

[26] Song Y, Lu M, Feng L, Chen Q, Huang H, Lin Q (2022). Identification of potential immunotherapy biomarkers for breast cancer by bioinformatics analysis. Biosci Rep.

[27] Zhuo C, Li X, Zhuang H, Tian S, Cui H, Jiang R (2016). Elevated THBS2, COL1A2, and SPP1 expression levels as predictors of gastric cancer prognosis. Cell Physiol Biochem.

[28] Song Z, Chen W, Athavale D, Ge X, Desert R, Das S (2021). Osteopontin takes center stage in chronic liver disease. Hepatology.

[29] Neubert NJ, Schmittnaegel M, Bordry N, Nassiri S, Wald N, Martignier C (2018). T cell-induced CSF1 promotes melanoma resistance to PD1 blockade. Sci Transl Med.

[30] Cui B, Fan X, Zhou D, He L, Li Y, Li D (2020). CSF1R methylation is a key regulatory mechanism of tumor-associated macrophages in hepatocellular carcinoma. Oncol Lett.

[31] Richardsen E, Uglehus RD, Johnsen SH, Busund LT (2015). Macrophage-colony stimulating factor (CSF1) predicts breast cancer progression and mortality. Anticancer Res.

[32] Zhu Y, Knolhoff BL, Meyer MA, Nywening TM, West BL, Luo J (2014). CSF1/CSF1R blockade reprograms tumor-infiltrating macrophages and improves response to T-cell checkpoint immunotherapy in pancreatic cancer models. Cancer Res.

[33] Wang H, Shao Q, Sun J, Ma C, Gao W, Wang Q (2016). Interactions between colon cancer cells and tumor-infiltrated macrophages depending on cancer cell-derived colony stimulating factor 1. Oncoimmunology.

[34] Katoh M (2016). FGFR inhibitors: Effects on cancer cells, tumor microenvironment and whole-body homeostasis (Review). Int J Mol Med.

[35] Barca C, Foray C, Hermann S, Herrlinger U, Remory I, Laoui D (2021). The colony stimulating factor-1 receptor (CSF-1R)-mediated regulation of microglia/macrophages as a target for neurological disorders (Glioma, Stroke). Front Immunol.

[36] Gao X, Jia G, Guttman A, DePianto DJ, Morshead KB, Sun KH (2020). Osteopontin links myeloid activation and disease progression in systemic sclerosis. Cell Rep Med.

