# Supplementary material for: Important oncogenic and immunogenic roles of SPP1 and CSF1 in hepatocellular carcinoma
Source: Med Oncol. 2023 Apr 25;40(6):158. doi: 10.1007/s12032-023-02024-7 (PMC10129977; doi:10.1007/s12032-023-02024-7)
Supplement: Supplementary file 1 — Supplementary file1 (DOCX 1043 KB) [file 12032_2023_2024_MOESM1_ESM.docx]

Supplementary Material

**Important oncogenic and immunogenic roles of SPP1 and CSF1**

**in hepatocellular carcinoma**

Tianxin Xiang ^1†^, Na Cheng ^1†^, Bo Huang^2^, Xujun Zhang^3^, Ping Zeng^4, 5*^

^1^ Department of Hospital Infection Control, The First Affiliated Hospital of Nanchang University, Nanchang, China.

^2^ Department of Gynecology and Obstetrics, The First Affiliated Hospital, Zhejiang University School of Medicine, Hangzhou, China.

^3^ Hangzhou Normal University School of Basic Medical Sciences, Hangzhou, China

^4^ Department of Radiology, Sir Run Run Shaw Hospital, Zhejiang University School of Medicine, No. 3, Qingchun East Road, Hangzhou, Zhejiang, China.

^5^ Department of Hospital Infection Control, The First Affiliated Hospital of Nanchang University, Nanchang, China.

*Correspondence:

Ping Zeng

**Address:** Department of Hospital Infection Control, The First Affiliated Hospital of Nanchang University. 17 Yongwai Road, Donghu District. Nanchang, China.

**Tel:** +86 15879193240

**Email:** ndyfy08004@ncu.edu.cn

† These authors have contributed equally to this work and share authorship.

**
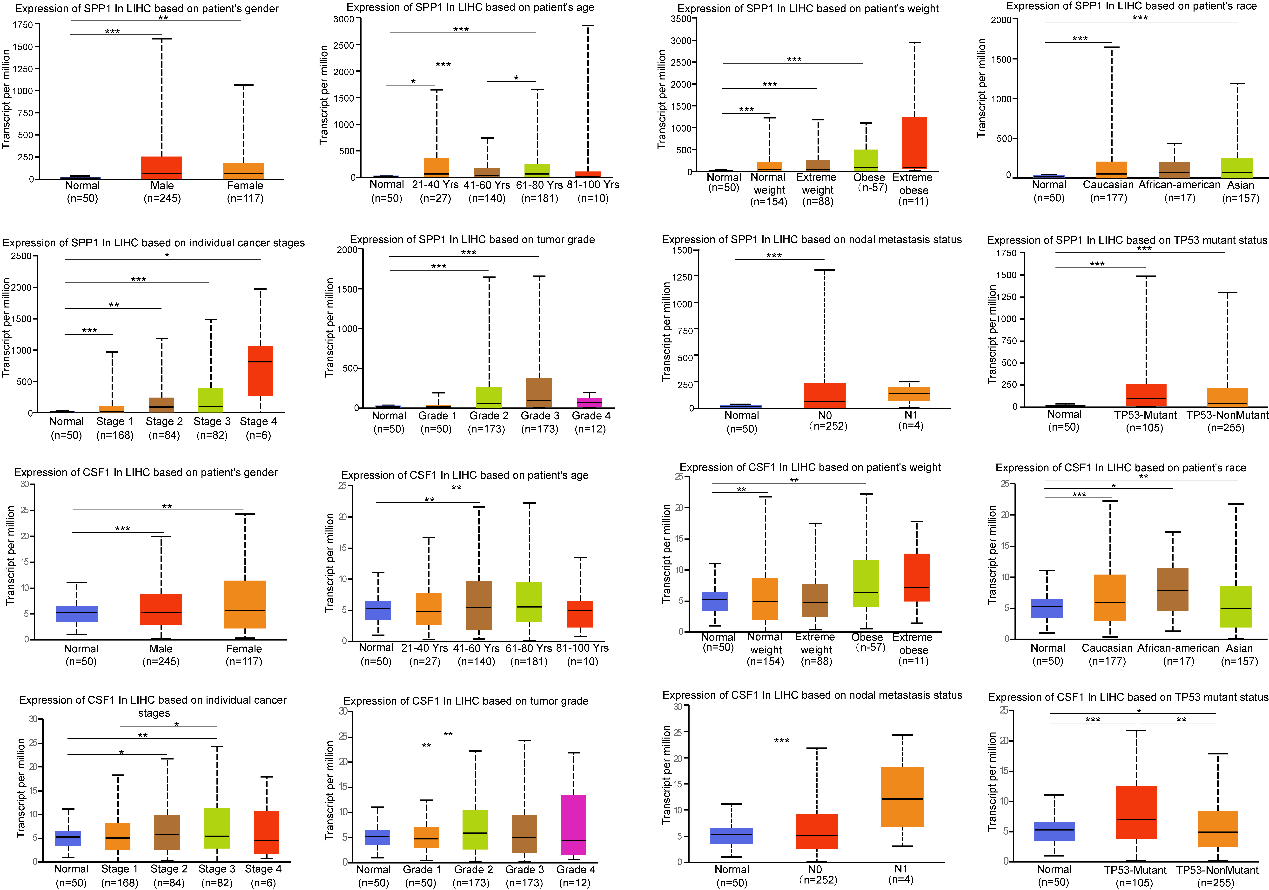
**

Figure S1 The expression levels of SPP1 and CSF1 in subgroup cancer samples. (*P<0.05, **P<0.01, ***P<0.001)


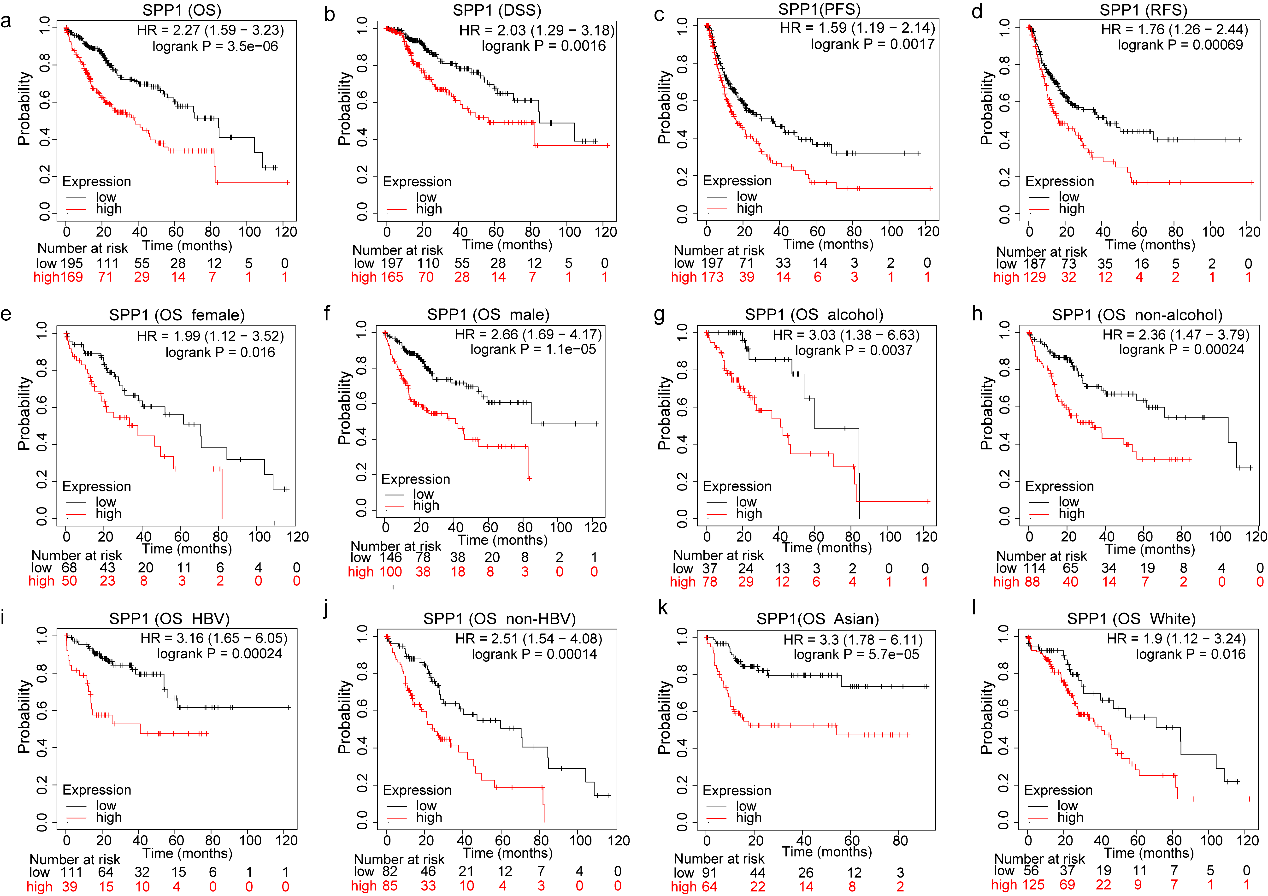


Figure S2 Survival analysis of SPP1. (a-d) Kaplan‒Meier survival curves displaying the correlation between SPP1 expression and OS, DSS, PFS, and RFS. (e-l) Kaplan‒Meier survival curves displaying the correlation between SPP1 expression and gender, race, HBV, and alcohol consumption.


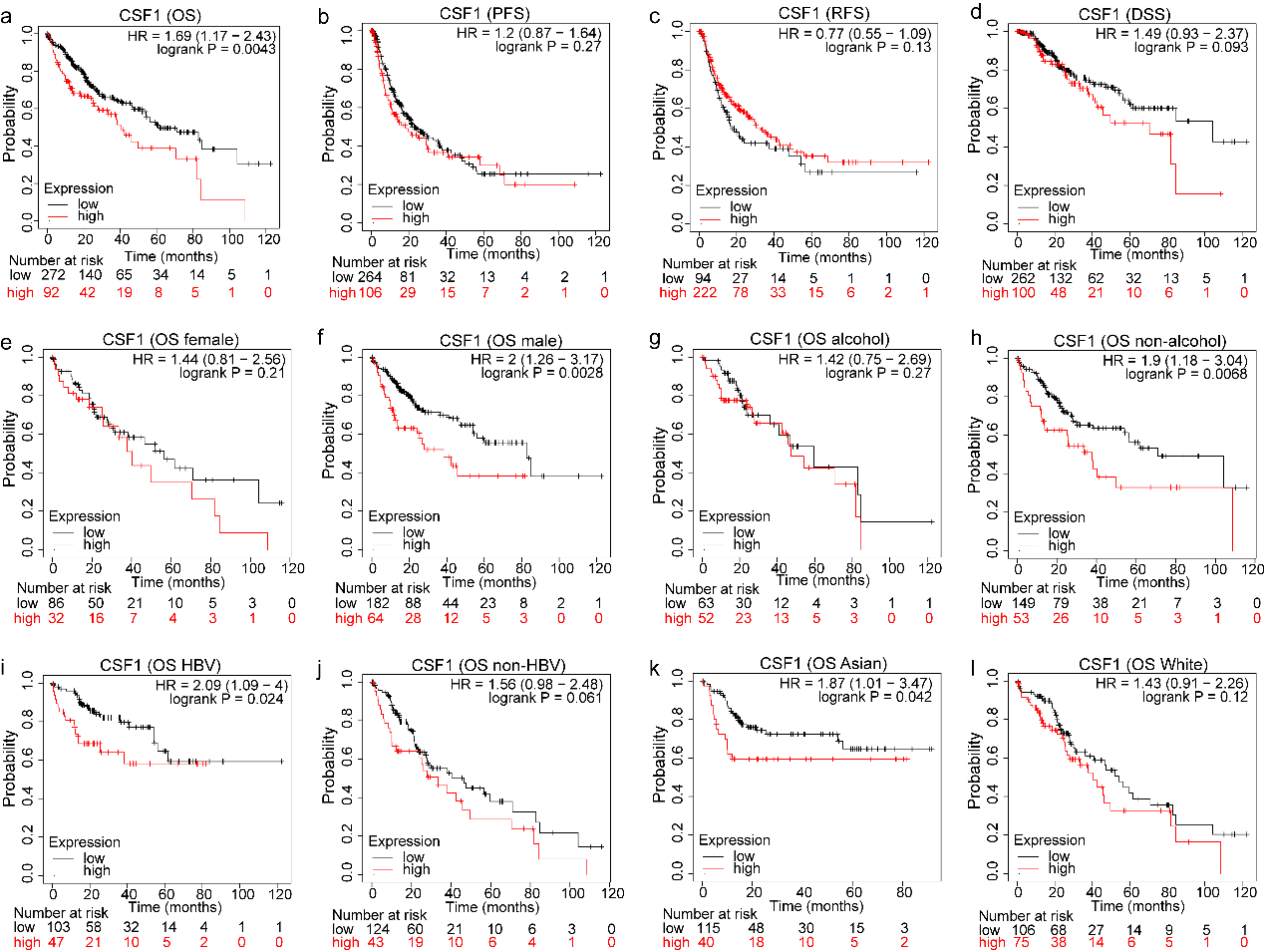


Figure S3 Survival analysis of CSF1. (a-d) Kaplan‒Meier survival curves displaying the correlation between CSF1 expression and OS, DSS, PFS, and RFS. (e-l) Kaplan‒Meier survival curves displaying the correlation between CSF1 expression and gender, race, HBV, and alcohol consumption.

**
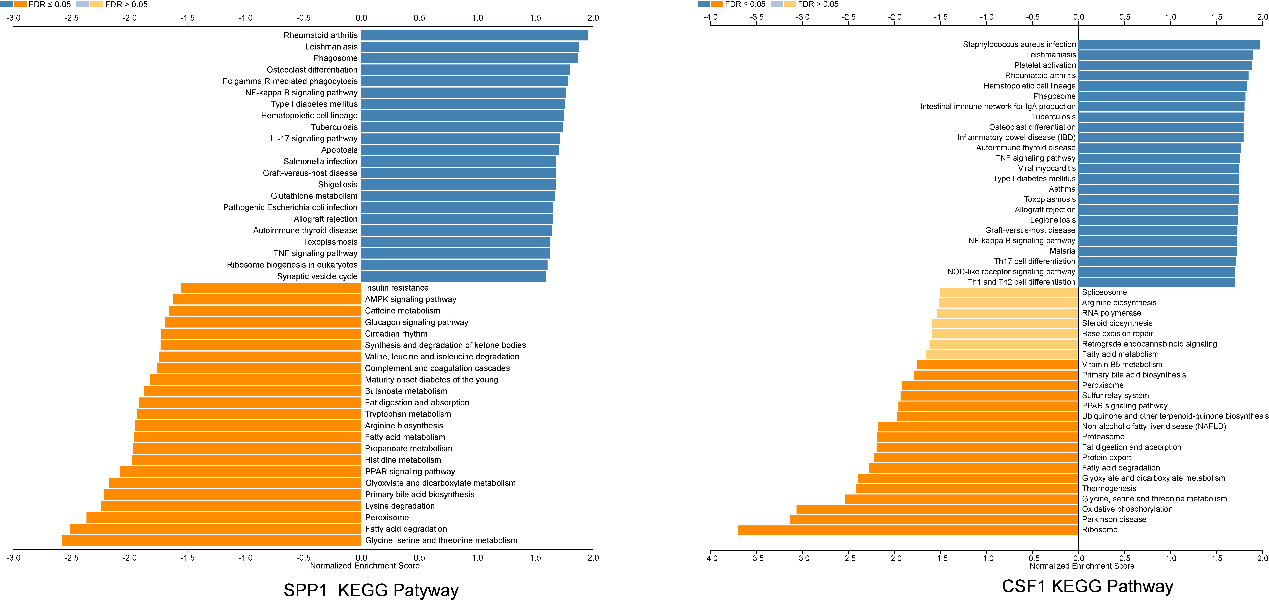
**

Figure S4 Signaling pathways involved in SPP1 and CSF1 were analyzed by LinkOmics database.


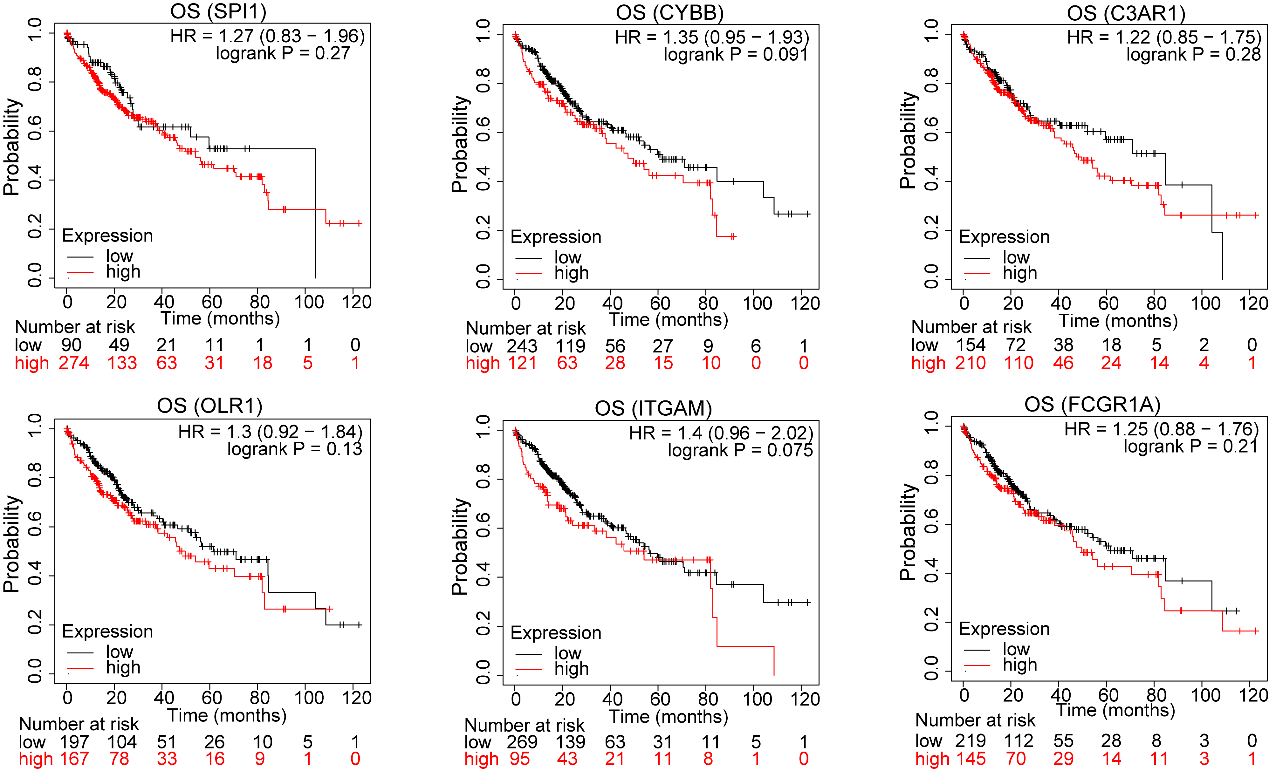


Figure S5 Survival analysis of six genes without statistically different including SPI1, FCGR1A, C3AR1, CYBB, OLR1, and ITGAM.
